# Sex Differences in How Territory Quality Affects Aggression in Convict Cichlids

**DOI:** 10.1093/iob/obab028

**Published:** 2021-10-18

**Authors:** Joseph M Leese, T Blatt

**Affiliations:** Department of Biology, DeSales University, 2755 Station Avenue, Center Valley, PA 18034, USA; Department of Biology, DeSales University, 2755 Station Avenue, Center Valley, PA 18034, USA

## Abstract

In animal contests, the value an individual assigns to limited resources can directly impact the level of aggression it demonstrates. For territorial species, individuals often assess their territory quality and appropriately modify the time and energy invested in its defense. In this study, male and female convict cichlids, *Amatitlania nigrofasciata*, were acclimated to one of three territorial treatments representing either a low, medium, or high resource value. Territories with a “Low Value” included substrate alone, “Medium Value” territories included substrate and a nest site, and a “High Value” territory included substrate, a nest site, and constant food source. After three days of acclimation, a size-matched intruder was introduced to elicit territorial aggression and behaviors were observed. Territory quality affected one measure of low-intensity aggression (displays) in residents but had no effect on high-intensity aggression (bites and chases). Moreover, there was a significant effect of sex, with males and females differing in the types of aggressive behaviors demonstrated across all treatments. Females showed more low-intensity aggressive behaviors toward intruders than males did. Additionally, a significant interaction of sex and territory quality was observed on two measures of high-intensity aggressive behavior (bites and chases), with females more likely than males to increase aggressive behaviors along with increasing territory quality. This suggests that females may be more sensitive and/or responsive to changes in the quality of a territory, possibly due to the necessity of a suitable nest site for egg deposition within a territory.

## Introduction

Aggressive behavior in animals is influenced by numerous factors, leading to tremendous variation among species, populations, and individuals in both the likelihood of engaging in aggression and the intensity of a given encounter. For territorial species, intraspecific aggression is common when suitable territory sites are limited and/or when available territories differ in quality, such as access to food, shelter, or potential mates (Brown 1964; Krebs 1982; Hinsch and Komdeur 2017). In the latter condition, levels of aggression have been shown to be influenced by the value (V) of the territory assigned by the resident animal; the greater the value, the more an individual is willing to fight or defend its territory. This has been demonstrated across a wide range of taxa, and numerous times in fishes specifically. In sand gobies, *Pomatoschistus minutus*, the size of a male's territory influences the level of aggression demonstrated toward a territorial intruder (Lindstrom and Pampoulie 2005). In brown trout, *Salmo trutta*, an individual's preference for a given substrate type within a territory influences its ability to successfully win contests (Johnsson et al. 2000). And in male beaugregory damselfish, *Stegastes leucostictus*, individuals provided with an artificially enhanced high-quality territory increased their aggression, while males that had territory quality reduced showed decreases in territorial aggression (Snekser et al. 2009).

While numerous empirical studies have documented the effects of resource value on aggressive behaviors, often, as in the examples just provided, this relationship is explored only within males. The focus on male, rather than female, aggression can likely be traced to the patterns of intrasexual selection first described by Darwin (1859). Males of many species must compete aggressively to secure territories or direct access to females for successful reproduction. From the female perspective, reproductive decisions are often thought of as primarily securing the highest quality male available through exercising choice rather than direct competition for resources (Andersson 1994). And while the patterns of heightened aggression in males compared with females may hold true for many species, especially those with polygynous and polygynandrous mating systems, this is far from universal. A growing number of studies have focused on female–female aggression and its underlying causes and consequences (Clutton-Brock 2009; Rosvall 2011; Cain and Ketterson 2012, 2013; Stockley and Campbell 2013; Cain 2014). Unsurprisingly, females show higher overall levels of aggression than males in sex-role reversed species, such as phalaropes and pipefish (Eens and Pinxten 2000). Females, however, can also show high levels of aggression in “sex-typical” species. This has been explored primarily in birds (Fedy and Stutchbury 2005; Heinsohn et al. 2005; Rosvall 2010), but also in lizards (Woodley and Moore 1999; Prosen et al. 2004; Wu et al. 2019), mammals (Bebié and McElligott 2006; Huchard and Cowlishaw 2011), and fish (Taves et al. 2009; Tubert et al. 2012). Many studies have focused on the ultimate pressures driving female–female aggression, as well as the underlying endocrinological mechanisms, with relatively less known about the ecological and environmental factors that might influence female–female aggression. For instance, in some monogamous species, both males and females can demonstrate aggression before and after forming pair bonds in non-territorial and territorial conflicts (Mackereth and Keenleyside 1993). When the conflict involves access to a limited territorial resource, the impact of territory quality on aggressive behavior may differ between the sexes if the fitness cost/benefit ratio of defending the territory is different for males and females.

Here, we explored whether sex differences might exist in how individuals alter their aggressive behavior due to variation in territory quality in a monogamous cichlid. Convict cichlids, *Amatitlania nigrofasciata*, are small freshwater fish native to rivers and lakes of Central America. During the breeding season, convict cichlids form monogamous pair bonds and work together to build a nest, defend a territory, and raise their offspring (Wisenden 1995). This species is well studied for their aggression in the field (McKaye 1977; Anderson et al. 2016; van Breukelen and Santangelo 2020) and the lab (Draud and Lynch 2002; Leiser et al. 2004; Arnott and Elwood 2009; Barley and Coleman 2010). While individuals in pair bonds cooperate in many tasks, studies have found significant differences in parental roles between males and females when fry are present, with males spending more time engaging in defense and females showing more direct interactions with young (Itzkowitz et al. 2005; Snekser et al. 2011).

In addition to sex differences in parental roles, Arnott and Elwood (2009) found that male and female convict cichlids use different fighting tactics as well. Males tend to use more lateral displays and tail beats, while females bite, chase, and use more frontal displays in intrasexual contests. However, Arnott and Elwood (2009) found no differences in the motivation to fight between the sexes when provided with similar environments, suggesting that the differences in fight tactics were not due to differences in the value placed on a specific resource, but some other intrinsic factor.

Given the sex differences that exist in parental roles as well as differences in specific behaviors used in aggressive encounters, we hypothesized that sex differences might also exist in the effect that the value of a territory has on aggressive behavior in this system. In the wild, pairs often form prior to selecting and defending a suitable territory. In the lab, however, single males and single females will defend a suitable territory from same-sex conspecific individuals if the situation is artificially created. We created territories for single individuals of both sexes and set out to alter territory value by the presence or absence of a nest site within a territory, as well as by supplementing territories with an abundant food source. Both the presence of nest sites and food availability have been shown to affect behavior in this species in certain contexts (Grant et al. 2002; Gumm and Itzkowitz 2007). Specifically, we predicted that territorial aggression would increase with the addition of a nest site and supplemental food source, and that males would show a greater difference in their behavioral response than females.

## Methods

Adult convict cichlids, *A. nigrofasciata*, were obtained from a combination of local pet suppliers and lab-raised stocks. The exact experience of fish prior to entering the lab is unknown, but once in the lab, all fish were housed in single-sex stock tanks (208 L) for at least two months prior to the start of the experiment. Stock tanks contained gravel substrate and a filtration system, and water temperature was maintained at 22 ± 2°C. The room was kept on a 14:10 h light:dark cycle and fish were fed commercially available pellet food once per day. All experimental protocols were approved by the IACUC at Muhlenberg College (Allentown, PA).

For the experimental treatments, three different territorial environments were created and labeled as Low, Medium, or High resource value (V). Territories were established using 75 L aquariums aerated by a single air stone and kept at 22 ± 2°C. The Low V territory consisted of gravel substrate only. The Medium V territory contained the same gravel substrate as well as a terra cotta flowerpot to serve as a nest site. The High V territory contained the gravel substrate, terra cotta flowerpot, and a commercially available slow release “food pyramid” (API). The food pyramid provides a continuous four-day supply of food for the fish and convict cichlids readily use this source of food in the lab (T.B., personal observation). All fish were fed pellet food once daily during the experiment and were not food deprived in any of the treatments. So, while the amount of food *per se* might not have contributed to the value of the food pyramid in the High V territory, we assumed that the constant availability of food might still increase the value to a territory holder.

At the start of each replicate, resident individuals were obtained from stock tanks and measured for total length (TL), standard length (SL), and mass (Table 1). Once measured, the subjects were assigned haphazardly to one of three treatments groups, transported to the appropriate experimental aquarium, and then given a 72 h acclimation period. After the acclimation, an intruder fish was obtained from stock tanks and measured in the same dimensions. Intruder fish were matched based on sex and size according to total length (+/−10%) with a resident fish (Table 1). Resident and intruder fish were housed separately at the end of each replicate to avoid pseudoreplication. The intruder was added directly to a territory with the size-matched resident and the ensuing contest was recorded using a digital video camera (JVC Model# GZ-MG630AU, California, USA) for 30 min. After the interaction, recorded contests were viewed, and a number of aggressive behaviors were counted. There was observable aggression between residents and intruders in all trials completed. Recorded behaviors included low-intensity aggression (displays) and high-intensity aggression (bites, chases, and mouth wrestling; for detailed descriptions of behaviors, see Itzkowitz et al. 2001). All behaviors were recorded as single events rather than states, and duration of displays or chases was not recorded, as any specific behavior rarely lasted for more than a few seconds. Behaviors were first combined for a measure of “total aggression” and later analyzed separately, except for mouth wrestling. There were only a few instances of mouth wrestling across all trials, so these were not analyzed separately, but they were included in the “total aggression” count. Ten trials were conducted with each sex for each of the three territory treatments. Two trials with female subjects (Medium V and High V treatments) were later discarded due to technical issues with the video recording (*N* = 58). Behavioral data were analyzed using a general linear model analysis of variance exploring the main effects of treatment and sex as well as interactions of the two factors. Due to the separate and repeated analyses of total aggression and then individual aggression measures, a Bonferroni correction was applied, and significance levels were set at *P* ≤ 0.025. In the case of a significant effect of treatment or a significant interaction of sex and treatment, Fisher's least significant difference (LSD) post-hoc tests were conducted for pairwise analyses. All analyses were conducted in SPSS 27. Data available from authors upon request.

**Table 1:** Size distributions (average ± standard error of the mean) for resident and intruder individuals across the three treatment groups for both sexes

	Resident			Intruder		
Treatment	TL (mm)	SL (mm)	M (g)	TL (mm)	SL (mm)	M (g)
**Male**						
Low V (*n* = 10)	84.4 ± 3.24	67.0 ± 2.90	10.3 ± 0.90	83.5 ± 3.28	67.0 ± 3.32	10.1 ± 0.96
Medium V (*n* = 10)	84.7 ± 2.53	68.7 ± 2.51	10.4 ± 0.91	84.8 ± 2.12	68.6 ± 2.14	9.9 ± 0.64
High V (*n* = 10)	86.7 ± 1.54	70.1 ± 1.39	11.6 ± 0.36	86.7 ± 1.65	70.0 ± 1.28	11.5 ± 0.53
**Female**						
Low V (*n* = 10)	76.1 ± 1.15	64.2 ± 1.52	6.5 ± 0.51	76.2 ± 2.07	63.6 ± 1.73	6.4 ± 0.58
Medium V (*n* = 9)	74.3 ± 2.79	61.3 ± 1.95	6.0 ± 0.66	73.7 ± 2.86	61.9 ± 2.08	5.7 ± 0.63
High V (*n* = 9)	74.2 ± 2.46	61.9 ± 1.90	5.6 ± 0.52	74.3 ± 2.55	61.5 ± 2.06	5.7 ± 0.60

## Results

**Fig. 1:**
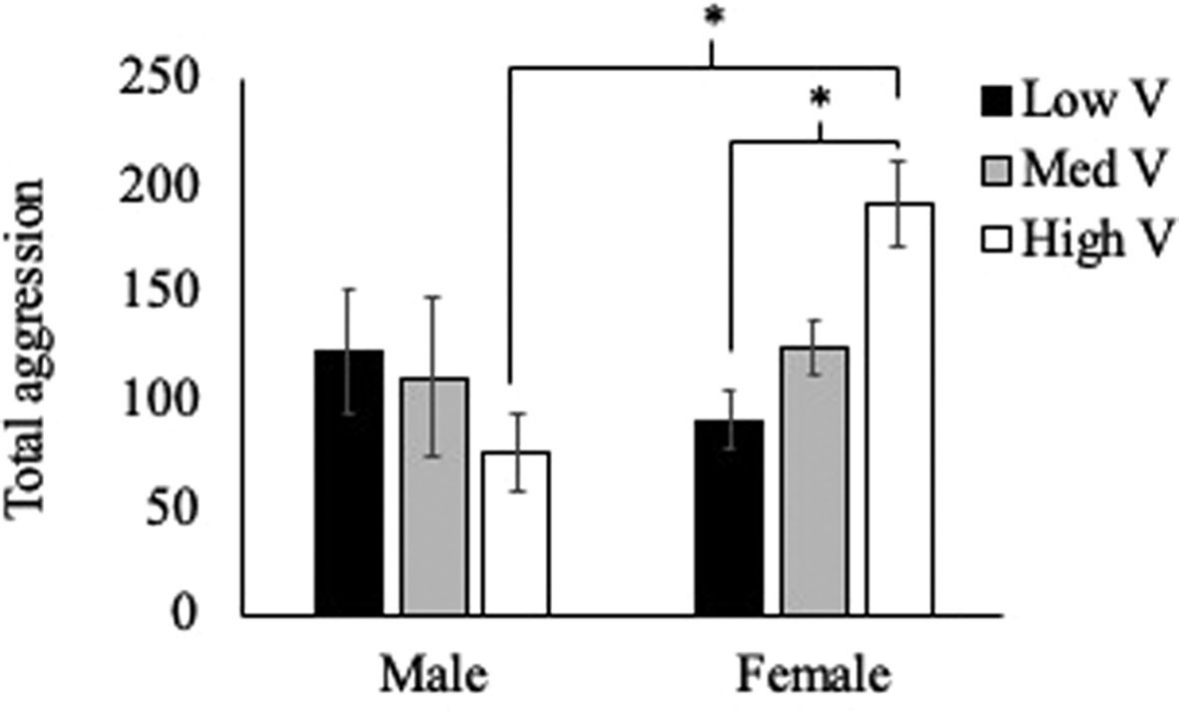
Average ± SEM total aggression (sum of all displays, bites, chases, and mouth wrestling) demonstrated by male and female residents across treatment groups. There were no main effects of treatment or sex; however, there was a significant interaction of treatment × sex on total aggression.

**Fig. 2:**
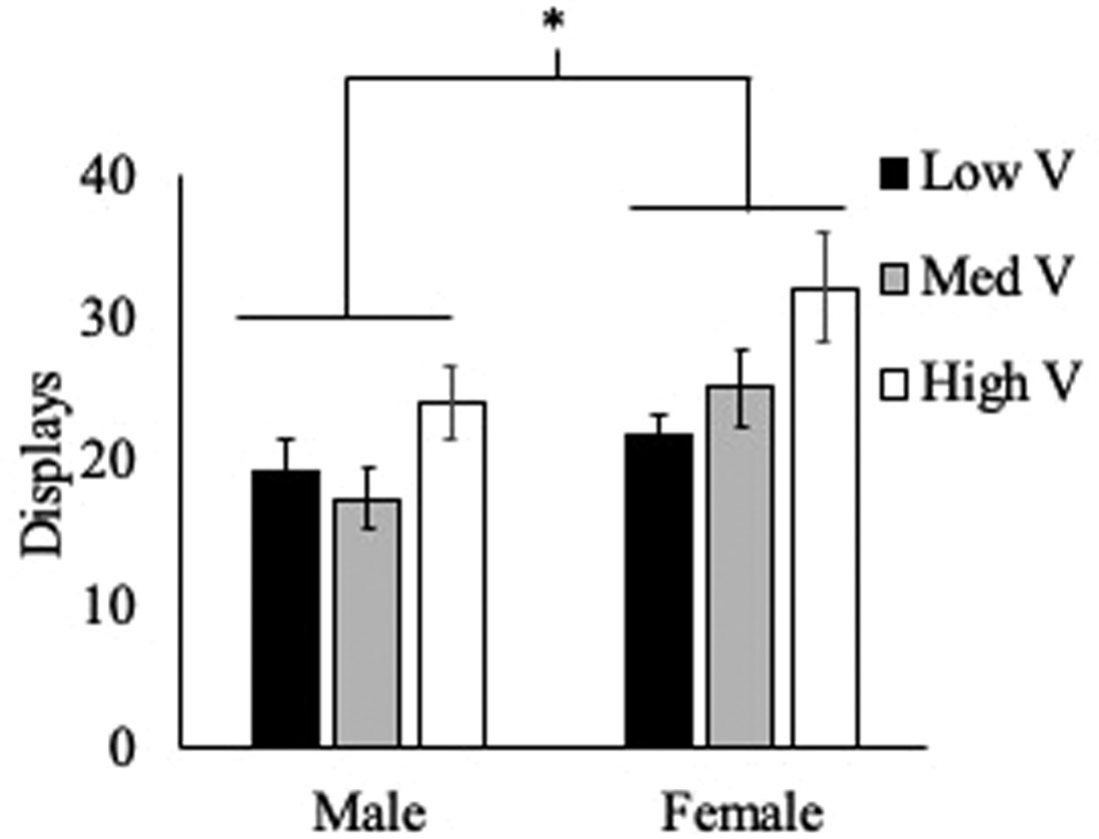
Average ± SEM displays, a form of low-intensity aggression, demonstrated by male and female residents across treatment groups. There were significant effects of treatment and sex, but no significant interaction of these factors on displays.

There was no significant effect of treatment (F_(2,57)_ = 0.638, *P* = 0.53) on total aggression demonstrated by residents. There was a nonsignificant trend of sex (F_(1,57)_ = 2.86, *P* = 0.09) impacting total aggression, and there was a significant interaction of these two factors (F_(2,57)_ = 4.994, *P* = 0.01; Fig. 1). When aggressive behaviors were classified as either low-intensity (displays) or high-intensity (chases and bites), more clear effects of treatment and sex appeared. For low-intensity aggression (displays), there was a significant effect of treatment (F_(2,57)_ = 5.561, *P* = 0.006) as well as sex (F_(1,57)_ = 9.196, *P* = 0.004). There was no significant interaction between these factors (treatment × sex, F_(2,57)_ = 0.788, *P* = 0.46; Fig. 2).

**Fig. 3:**
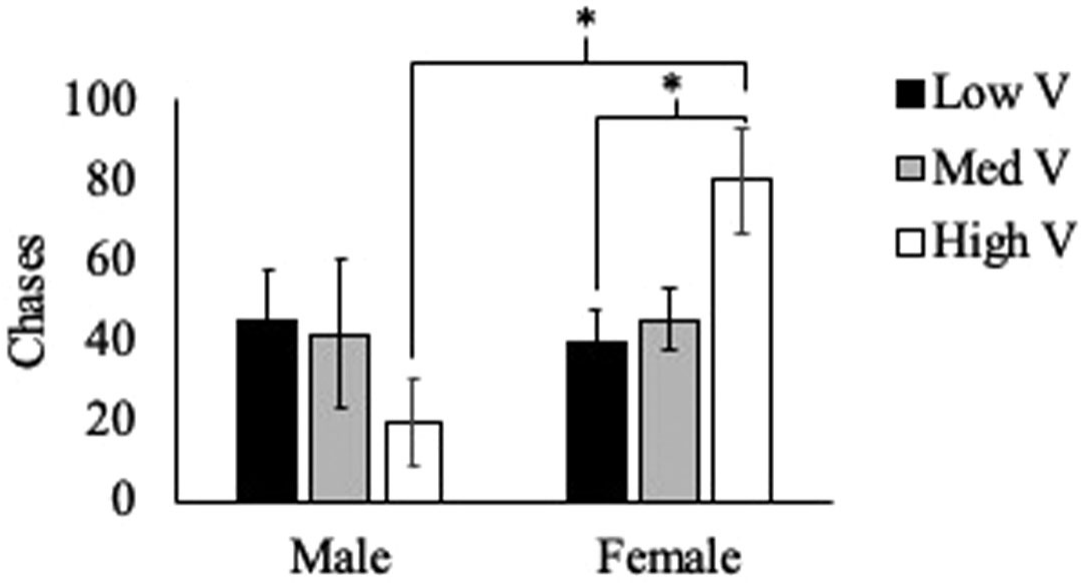
Average ± SEM chases, a form of high-intensity aggression, demonstrated by male and female residents across treatment groups. There was no significant effect of treatment or sex; however, there was a significant interaction of these factors on chases.

**Fig. 4:**
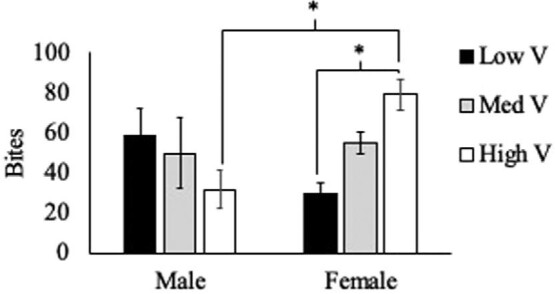
Average ± SEM number of bites, a form of high-intensity aggression, demonstrated by male and female residents across treatment groups. There was no significant effect of treatment or sex; however, there was a significant interaction of these factors on bites.

Two measures of high intensity aggression showed the same patterns regarding the effects of treatment and sex. There were no significant effects of treatment (F_(2,57)_ = 0.208, *P* = 0.81) or sex (F_(1,57)_ = 3.553, *P* = 0.06) on chases (Fig. 3) or effects of treatment (F_(2,57)_ = 0.552, *P* = 0.58) or sex (F_(1,57)_ = 0.731, *P* = 0.40) on bites (Fig. 4), but there were significant interactions of these variables on both chases (F_(2,57)_ = 3.947, *P* = 0.025) and bites (F_(2,57)_ = 5.757, *P* = 0.006).

**Fig. 5:**
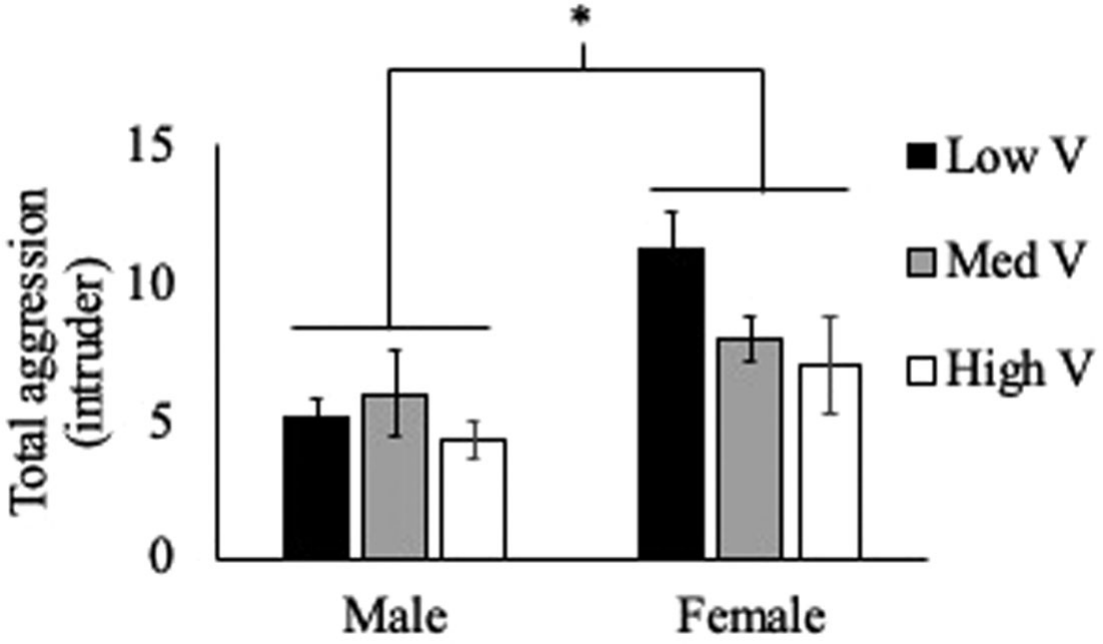
Average ± SEM total aggressive behaviors demonstrated by male and female intruders across treatment groups. There was no significant effect of treatment, but there was a significant effect of sex. Female intruders showed higher levels of aggression than male intruders. There was no significant interaction of these two factors on aggression.

Behavior of the intruder individuals was also recorded and analyzed. There was no effect of treatment on any behaviors of the intruder (total aggression: F_(2,57)_ = 0.655, *P* = 0.52; displays: F_(2,57)_ = 0.943, *P* = 0.40; chases: F_(2,57)_ = 1.074, *P* = 0.35; bites: F_(2,57)_ = 1.185, *P* = 0.31), but there was a significant effect of sex on total aggression (F_(1,57)_ = 21.928, *P* < 0.001), bites (F_(1,57)_ = 8.733, *P* = 0.005), and displays (F_(1,57)_ = 40.187, *P* < 0.001), but not chases (F_(1,57)_ = 0.262, *P* = 0.61). There was no significant interaction between treatment and sex on any intruder behaviors (total aggression: F_(2,57)_ = 1.755, *P* = 0.18; displays: F_(2,57)_ = 1.233, *P* = 0.30; chases: F_(2,57)_ = 0.644, *P* = 0.53; bites: F_(2,57)_ = 0.669, *P* = 0.52). Only the data for total aggression by intruders are presented here (Fig. 5).

For measures with significant main effects of treatment (displays) or a significant interaction (total aggression, bites, and chases), pairwise comparisons were made using Fisher's LSD post-hoc tests where appropriate. For behaviors affected by significant interactions of treatment and sex, pairwise comparisons were made within each sex across the treatment groups (i.e., Male High V vs. Male Med V) as well as between the sexes within each treatment group (i.e., Male High V vs. Female High V). Pairs that differed in both sex and treatment were not directly compared (i.e., Male High V vs. Female Low V). Post-hoc tests were not necessary for significant effects of sex due to only having two levels of the variable. The summary of these pairwise comparisons is presented in Table 2.

**Table 2:** Pairwise comparisons using Fisher's LSD post-hoc tests for behaviors with significant main effects of treatment (displays) or interactions of treatment × sex (total aggression, bites, and chases). Significant levels of *P* ≤ 0.05 are indicated in bold

Significant main effects	Pairwise comparison	*P*
**Treatment**		
Displays	High V vs. Med V	**0.009**
	High V vs. Low V	**0.005**
	Med. V vs. Low V	0.840
**Treatment × sex**		
Total aggression	Male High V vs. Male Med V	0.245
	Male High V vs. Male Low V	0.094
	Male Med V vs. Male Low V	0.598
	Female High V vs. Female Med V	0.148
	Female High V vs. Female Low V	**0.003**
	Female Med V vs. Female Low V	0.125
	Male High V vs. Female High V	**0.005**
	Male Med V vs. Female Med V	0.767
	Male Low V vs. Female Low V	0.073
Bites	Male High V vs. Male Med V	0.245
	Male High V vs. Male Low V	0.094
	Male Med V vs. Male Low V	0.598
	Female High V vs. Female Med V	0.148
	Female High V vs. Female Low V	**0.003**
	Female Med V vs. Female Low V	0.125
	Male High V vs. Female High V	**0.005**
	Male Med V vs. Female Med V	0.767
	Male Low V vs. Female Low V	0.073
Chases	Male High V vs. Male Med V	0.213
	Male High V vs. Male Low V	0.158
	Male Med V vs. Male Low V	0.865
	Female High V vs. Female Med V	0.068
	Female High V vs. Female Low V	**0.029**
	Female Med V vs. Female Low V	0.735
	Male High V vs. Female High V	**0.002**
	Male Med V vs. Female Med V	0.841
	Male Low V vs. Female Low V	0.756

## Discussion

Contrary to our initial hypothesis, we did not see an increase in total aggressive behavior as the value of the territory increased. We also found no support for our hypothesis that males would show higher levels of aggression compared with females and that they would be more responsive to the changes in territory quality. The data actually support the opposite pattern. While sex did not have a significant impact on overall aggression, there was a nonsignificant trend (*P* = 0.09) suggesting that females demonstrated more aggression overall than males across the three treatments. And this pattern was significant (*P* = 0.004) when focusing exclusively on low-intensity aggression. Females showed significantly more displays than males, and this difference appears to be driven by the increase in aggression that females showed in the High V territory treatment. The significant interaction of sex and treatment was present on both low-intensity and high-intensity aggression, as well as total aggression, with males demonstrating similar levels of behavior across the three territory value treatment groups, while females showed an increase in aggression correlating with increases in territory quality, as we predicted.

While unexpected, these findings are not entirely unique when considering sex differences in aggressive behavior, especially in fishes. As stated previously, Arnott and Elwood (2009) reported differences in fight tactics in convict cichlids, and this study corroborated those findings. The authors did not, however, find the sex differences in aggression were due to differences in motivation, and here the differences do seem to be driven by the value placed on the territory treatment itself. Another study in this system also found that sex differences in aggression could be driven by external factors. Specifically, males and females responded differently in aggressive behavior after the loss of a pair-bonded partner (van Breukelen and Itzkowitz 2011). Males showed a greater increase in aggression toward intruders after losing a partner than females did in the same situation. This study, however, focused on aggression within a parental defense context and was conducted with the presence of fry. The influence of sex-specific parental care roles likely contributed to differences in aggressive behavior between males and females. Additionally, studies in the distantly related Texas cichlid, *Herichthys cyanoguttatum*, have shown a similar pattern that males and females differ in both tactics in individual contests and aggression when guarding fry (Itzkowitz 1985; Draud et al. 2003).

In this experiment, the intrasexual contests occurred outside of a pair-bonding and reproductive context, in so far as a potential partner was not present for the contest. Therefore, the differences in aggression should not have been directly impacted by the biparental division of labor seen in this species (Itzkowitz et al. 2001, 2002; Snekser and Itzkowitz 2020) or direct competition for potential mates (Bloch et al. 2016). This then begs two specific questions: (1) Why were females generally more aggressive than males? and (2) Why do females seem to be more sensitive to the changes in territory quality than males?

As far as the first question is concerned, there are notable other species where females demonstrate higher aggression levels when compared with males. In black chin tilapia, *Sarotherodon melanotheron*, females demonstrate more aggression than males in competition for mates (Balshine-Earn and McAndrew 1995). This is also true in other cichlids (Wood et al. 2014; Ito et al. 2017) as well as pipefish (Berglund et al. 2005; Rosenqvist 2009) and gobies (Swenson 1997; Amundsen 2018). However, all of these systems are generally considered “sex-role reversed,” due to males providing significant parental investment through paternal care of eggs and/or young (Trivers 1972) and the high levels of aggression are observed within the context of competing for mates. While there is a degree of ambiguity in assigning a species as “sex-role reversed” (see Barlow 2005), convict cichlids do not generally meet these criteria as both sexes demonstrate what are considered “sex-typical” roles in providing parental care. Females tend to provide more direct care of the young as both eggs and fry, and males provide more indirect care through aggressive defense. Convict cichlids do, however, demonstrate at least one similarity with sex-role reversed species. While males demonstrate sex-typical larger body size within bonded pairs (Wisenden 1995), females show greater ornamentation than males, which is a rare occurrence absent of sex-role reversal (but see Heinsohn et al. 2005). Females of this species develop bright orange ventral coloration, which has been shown to influence intrasexual aggression (Beeching et al. 1998; Anderson et al. 2016).

Yet regardless of whether the species has some features of sex-role reversal, the aggression that was observed here was in the context of a territorial intruder and not in the context of directly competing for access to mates. This leaves the ultimate selection pressures causing higher aggression in females than males still unexplained. One possible explanation is that females place a higher value on holding a territory in this system than males. While in natural conditions males have been shown to secure territories prior to forming pair bonds, and females have not, the requirement of a suitable nest site for egg deposition might have led females to more aggressively defend a territory once secured. Another possibility is that males have evolved a better ability to assess the outcome of a given aggressive encounter than females. The selection on males to more accurately assess their own likelihood of winning or losing a contest might be driven primarily by interactions in their role of defending offspring during the period of parental care, but this ability could spill over into other contexts such as a territorial dispute. Thus, territorial encounters between two males might not escalate in the same way they would with females, resulting in females showing higher levels of aggression than males. This is entirely speculative but supported at least in concept by the fact that female intruders also showed higher levels of aggression than male intruders, and this was completely independent of the territory treatment. Future studies would be needed to determine whether females are less able to accurately predict the outcome of a contest than males in this system, resulting in more escalation.

As far as the second question is concerned, we assume that the specific way in which we increased territory quality in this experiment could potentially have more of a direct impact on how a female values the territory than it would on a male. The first addition to increase the territory quality was the presence of a nest site. While a requirement for both members of a monogamous pair prior for reproduction, females seem to place a high value on the presence of a nest site in reproductive decisions, but this is unclear in males of this species (Gagliardi-Seeley et al. 2021). Given that females directly oviposit adhesive eggs to the surface of the nest site, it might be expected that they have experienced stronger selection pressure on finding a suitable substrate to serve as a nest site than males. In a similar manner, we also increased territory quality by adding an abundant food source. As neither males nor females were food deprived in this experiment, the additional food source may not have provided much incentive to increase defense of the territory. However, energy availability in general has the potential to directly influence female fecundity much more than males, and females may have been more sensitive to the increase in food availability.

In summary, we found that male and female convict cichlids respond differently in aggressive behavior with changes in the value of a territory. Females tend to show more aggression and moderate their aggression levels based on the value of a given territory, while males seemed indifferent to the changes in territory quality. These differences likely reflect basic sex differences in behavior and physiology, as well as the differing selection pressures the sexes have experienced on how the external environment affects their direct fitness. Future research could build on previous studies in other fishes that explore the proximate mechanisms regulating sex differences in aggression, and specifically what role is played by steroid hormones (Desjardins et al. 2006; Taves et al. 2009; Tubert et al. 2012). When placed into the larger context of research within this model system, this study supports the finding that stereotypical sex roles in behavior are more nuanced than might be expected based on mating system and reproductive strategies alone.
